# Supplementary material for: Effect of Age and Gender on the Efficacy of a 12-Month Body Weight Reduction Program Conducted Online—A Prospective Cohort Study
Source: Int J Environ Res Public Health. 2022 Sep 22;19(19):12009. doi: 10.3390/ijerph191912009 (PMC9566561; doi:10.3390/ijerph191912009)
Supplement: Supplementary file 1 [file ijerph-19-12009-s001.zip › ijerph-1904845-supplementary.pdf]

Table S1. Changes in body weight in gender groups over 12 months.

Body mass	Men (n=190)					Women (n=210)					
	**Mean ±SD [kg]**	**Median (min-max) [kg]**	**Change [%]**	**Change [kg]**	**P***	**Mean ±SD [kg]**	**Median (min-max) [kg]**	**Change [%]**	**Change [kg]**	**P***	**P****
Start	103,6 ± 16,02	103 (64,8-156,8)	-	-	-	89,1 ± 14,8	87 (63-142)	-	-	-	-
In 1 month	101,4 ± 15,47	101 (62,2-151,8)	-2,2	-2,2		87,3 ± 14,6	84,7 (62-140)	-2,0	-1,8		0,49
In 2 month	99,7 ± 15,1	99,2 (60,6-148,2)	-1,6	-1,7		85,8 ± 14,4	82,1 (61-138)	-1,7	-1,5		0,06
In 3 month	98,1 ± 15,1	98 (59,3-145,1)	-1,6	-1,6		84,4 ± 14,1	81 (60,2-136)	-1,7	-1,4		0,06
In 4 month	96,8 ± 14,8	97 (57,5-143,2)	-1,3	-1,3		82,9 ± 14,1	80,5 (58,2-134)	-1,7	-1,5		0,00001
In 5 month	95,4 ± 14,7	94,4 (56,2-142,1)	-1,4	-1,4		81,5 ± 13,9	79 (57-132)	-1,7	-1,4		0,00001
In 6 month	94 ± 14,5	92,1 (54-140,1)	-1,4	-1,4		80,1 ± 13,6	78 (55-130)	-1,8	-1,4		0,00001
In 7 month	92,8 ± 14,2	91,1 (52,3-139,1)	-1,3	-1,2	0,0001	78,8 ± 14,0	76 (54-129,3	-1,7	-1,3	0,0001	0,00001
In 8 month	91,7 ± 14,1	90,1 (51,8-136)	-1,3	-1,1		77,6 ± 14,0	74 (54,1-128,5)	-1,5	-1,2		0,00001
In 9 month	90,9 ± 13,9	89,4 (51-134,2)	-1,0	-0,8		76,5 ± 14,1	73 (53,7-128)	-1,5	-1,1		0,00001
In 10 month	90,0 ± 13,2	88,5 (50,2-136)	-1,0	-0,9		75,4 ± 14,3	72 (53-127)	-1,5	-1,1		0,00001
In 11month	89,2 ± 13,3	88 (50-134,2)	-0,9	-0,7		74,4 ± 14,4	70,9 (51,1-126)	-1,4	-1,0		0,00001
In 12 month	88,4 ± 13,1	87 (50-133,1)	-1,0	-0,8		73,7 ± 14,4	69,8 (50-124,5)	-0,9	-0,7		0,51
After 12 months	88,4 ± 13,1	68,7 (49,8-95)	-14,7	-15,2		73,7 ± 14,4	69,8 (50-124,5)	-17,4	-15,4		0,00001

* Significance of the change in body weight compared to the previous month – Friedman's rank test;
**Mann-Whitney U Test - Diference beetwen group.

Table S2. Changes in body weight in the 3 age groups over 12 months.

Body mass	Person in age 18-29 (n=136)					Person in age 30-39 (n=156)					Person in age 40-55 (n=108)					
	**Mean ±SD [kg]**	**Median (min-max) [kg]**	**Change [%]**	**Change [kg]**	**P***	**Mean ±SD [kg]**	**Median (min-max) [kg]**	**Change [%]**	**Change [kg]**	**P***	**Mean ±SD [kg]**	**Median (min-max) [kg]**	**Change [%]**	**Change [kg]**	**P***	**P****
Start	92,9 ± 15,6	90,6 (65-133)	-	-	-	97,1 ± 17,9	95 (63-156,8)	-	-	-	98,3 ± 16,9	97,9 (67-142,6)	-	-	-	-
In 1 month	91 ± 15,2	89 (64-130)	-2,0	-1,9		95,1 ± 17,4	92,6 (62-151,8)	-2,0	-2,0		96,3 ± 16,6	95,6 (64,2-140)	-2,1	-2		0,93
In 2 month	89,4 ± 14,9	77,8 (63-127)	-1,7	-1,6		93,4 ± 17,1	91 (60,2-150)	-1,7	-1,7		94,8 ± 16,4	93,5 (62,1-139)	-1,6	-1,5		0,9
In 3 month	88 ± 14,7	86 (62,1-126)	-1,5	-1,4		91,9 ± 17,0	90,1 (59,3-146)	-1,6	-1,5		93,3 ± 16,1	92,7 (60,2-138)	-1,6	-1,5		0,98
In 4 month	86,5 ± 14,7	85 (61,7-125)	-1,6	-1,5		90,5 ± 16,9	89,1 (57,5-144)	-1,6	-1,4		91,6 ± 16,1	91 (58,3-137)	-1,8	-1,7		0,41
In 5 month	85,3 ± 14,5	84 (60-125)	-1,5	-1,2		89,2 ± 16,8	88 (56,2-142)	-1,5	-1,3		90,2 ± 16,1	89,5 (57-136)	-1,6	-1,4		0,42
In 6 month	83,9 ± 14,4	82 (59-124)	-1,7	-1,4		87,8 ± 16,7	87 (54-141,1)	-1,6	-1,4		88,9 ± 16,1	89 (57-135)	-1,5	-1,3		0,11
In 7 month	82,7 ± 14,4	81 (58-123)	-1,5	-1,2	0,0001	86,5 ± 16,6	86 (52,3-137)	-1,5	-1,3	0,0001	87,6 ± 16,1	88 (56-133,6)	-1,5	-1,3	0,0001	0,47
In 8 month	81,5 ± 14,4	79,8 (57-123)	-1,5	-1,2		85,3 ± 16,5	85 (51,8-136)	-1,4	-1,2		86,6 ± 16,0	86,1 (54,1-132)	-1,3	-1,0		0,2
In 9 month	80,5 ± 14,3	78,5 (56-122,7)	-1,2	-1		84,2 ± 16,5	84 (51-135)	-1,4	-1,1		85,6 ± 16,0	85,1 (54-131)	-1,3	-1,0		0,06
In 10 month	79,5 ± 14,2	77,2 (55-122)	-1,4	-1		83,2 ± 16,5	82,5 (50,2-136)	-1,2	-1,0		84,6 ± 15,9	84 (53-130,2)	-1,3	-1,0		0,35
In 11 month	78,6 ± 14,2	77 (54,1-121)	-1,3	-0,9		82,3 ± 16,5	82 (50-137)	-1,1	-0,9		83,8 ± 16,4	83,4 (51,1-130)	-1,0	-0,8		0,07
In 12 month	77,8 ± 14,1	76,4 (53-120,8)	-1,0	-0,8		81,7 ± 16,4	81 (49,8-134)	-0,7	-0,6		83,0 ± 16,9	92,9 (50,1-130)	-1,0	-0,8		0,95
After 12 months	77,8 ± 14,1	76,4 (53-120,8)	-16,5	-15,1		81,7 ± 16,4	81 (49,8-134)	-16,0	-15,4		83,0 ± 16,9	92,9 (50,1-130)	-15,9	-15,3		0,14

* Significance of the change in body weight compared to the previous month – Friedman's rank test;
**Kruskal-Wallis Test - Diference beetwen group
